# CAPE Analogs Induce Growth Arrest and Apoptosis in Breast Cancer Cells

**DOI:** 10.3390/molecules200712576

**Published:** 2015-07-10

**Authors:** Annie-Pier Beauregard, Jason Harquail, Grégoire Lassalle-Claux, Mehdi Belbraouet, Jacques Jean-Francois, Mohamed Touaibia, Gilles A. Robichaud

**Affiliations:** 1 Department of Chemistry and Biochemistry, Université de Moncton, Moncton, NB E1A 3E9, Canada; E-Mails: eab7744@UMoncton.ca (A.-P.B.); ejh8189@umoncton.ca (J.H.); lassalleclaux.gregoire@gmail.com (G.L.-C.); mehdi.b1994@gmail.com (M.B.); jacques.jean-francois@umoncton.ca (J.J.-F.); mohamed.touaibia@umoncton.ca (M.T.); 2 Atlantic Cancer Research Institute, Moncton, NB E1C 8X3, Canada

**Keywords:** breast cancer, CAPE, apoptosis, NFκB, p53, caspase

## Abstract

Breast cancer is the second leading cause of death amongst women worldwide. As a result, many have turned their attention to new alternative approaches to treat this disease. Caffeic acid phenylethyl ester (CAPE), a well-known active compound from bee propolis, has been previously identified as a strong antioxidant, anti-inflammatory, antiviral and anticancer molecule. In fact, CAPE is well documented as inducing cell death by inhibiting NFκB and by inducing pro-apoptotic pathways (*i.e.*, p53). With the objective of developing stronger anticancer compounds, we studied 18 recently described CAPE derivatives for their ability to induce apoptosis in breast cancer cell lines. Five of the said compounds, including CAPE, were selected and subsequently characterised for their anticancer mechanism of action. We validated that CAPE is a potent inducer of caspase-dependent apoptosis. Interestingly, some newly synthesized CAPE derivatives also showed greater cell death activity than the lead CAPE structure. Similarly to CAPE, analog compounds elicited p53 activation. Interestingly, one compound in particular, analog **10**, induced apoptosis in a p53-mutated cell line. These results suggest that our new CAPE analog compounds may display the capacity to induce breast cancer apoptosis in a p53-dependent and/or independent manner. These CAPE analogs could thus provide new therapeutic approaches for patients with varying genotypic signatures (such as p53 mutations) in a more specific and targeted fashion.

## 1. Introduction

Breast cancer is still one of the deadliest cancers among women worldwide. Mechanistically, multiple genetic alterations foster the development and progression of cancer cells. For example, the activation of oncogenes and the alteration of tumor suppressor gene pathways are conducive to the development of neoplastic tissues. Essentially, cancer cells lose complete control over highly-regulated cell growth signals, which results in aberrant proliferation while evading programmed cell death or apoptosis [1].

A pivotal regulator of cell growth and survival is the NFκB transcription factor. The deregulation of NFκB is considered the cancer cell’s green light for proliferation and hyperactivity due to its anti-apoptotic effects [2,3]. Mechanistically, phosphorylation of the NFκB inhibitor (IκB) by IκB kinases (IKK) enables NFκB nuclear translocation and induction of pro-survival gene expression [4,5]. Another prominent cancer cell regulator working in concert with NFκB is the tumor suppressor gene p53. Studies have shown that the pro-apoptotic p53 gene is in fact inactivated or mutated in approximately 64% of all human cancers (reviewed in [6]). When irreparable genome damage occurs, p53 leads to the induction of the mitochondrial-mediated apoptotic pathway through the activation of pro-apoptotic genes such as Bax and p21 [7,8,9]. The importance of NFκB and p53 in cell homeostasis is reflected by the ongoing research interests and studies of NFκB and p53 as anticancer strategies [10,11,12].

**Figure 1:**
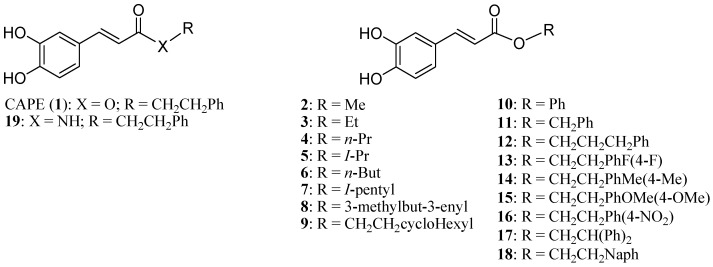
Caffeic Acid Phenethyl Ester (CAPE) derivatives.

The alarming incidence of cancer-related mortality has consistently pushed research toward the identification and development of new effective anticancer therapeutic strategies. A promising source for anticancer drug discovery is the use of bioactive compounds from natural products (reviewed in [13,14]). Phytochemicals such as polyphenols, flavonoids and phenolic acids have garnered wide interest by the scientific community due to their specific interactions with biological targets [15,16]. More specifically, caffeic acid phenylethyl ester (CAPE) (**1**, Figure 1), a prominent plant phenolic acid, has generated significant attention because of its ability to elicit cancer cell death mainly through the suppression of the cell’s survival pathway (*i.e.*, NFκB) [17,18,19] or concomitant induction of apoptosis cascades (*i.e.*, Bax, p53, *etc.*) [18,20,21,22]. Here we present the design of novel CAPE analogs and their capacity to modulated pivotal cell fate signaling cascades resulting in the apoptosis of breast cancer cell models.

## 2. Results and Discussion

### 2.1. Biological Evaluation of CAPE Analogs on Breast Cancer Cells

**Figure 2:**
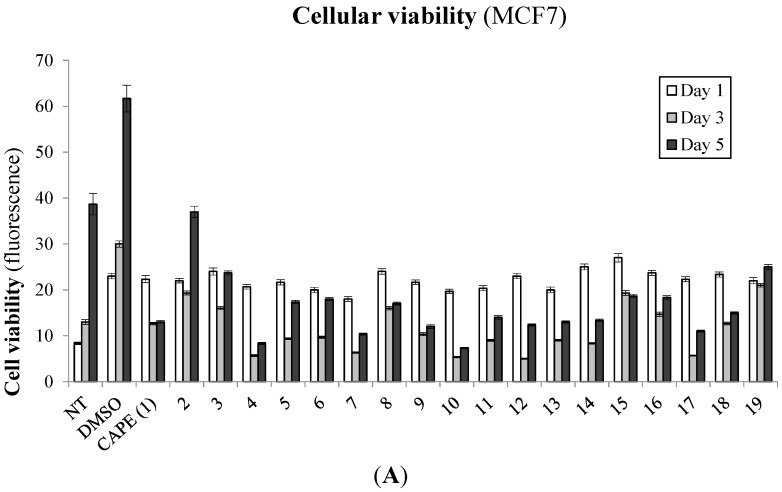

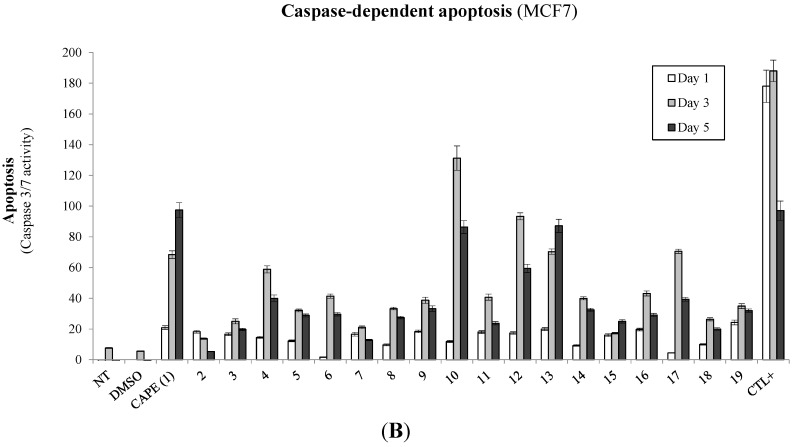
Biological effects of CAPE derivatives on breast cancer cells. MCF7 breast cancer cells were treated with 10 µM of selected compounds and incubated in time (24, 72 and 120 h) and evaluated for (**A**) cell viability assay (Cell-Titer Blue, Promega) or (**B**) caspase-dependent apoptotic events (Apo-ONE, Promega). Control samples include non-treated (NT) and solvent (DMSO) treated cells in addition to cells treated with apoptosis-inducing agent Melphalan (5 µM) as a positive control (CTL+). Results and standard deviations are representative of biological and experimental triplicates where the data is presented as the means ± SEM.

To establish the biological significance of our novel CAPE derivatives on cancer cell viability, we tested growth inhibition following treatment of CAPE (**1**) derivatives on the MCF7 non-aggressive breast cancer cell line. The cells were treated with 10 µM of the compounds and monitored for cellular viability at 1, 3 and 5 days post-treatment (Figure 2A). As expected, CAPE (**1**) showed strong growth inhibition within 48 h of treatment and suppressed up to 88% of growth when compared to the DMSO solvent control samples. Interestingly, many of the CAPE derivatives also showed a similar, if not more effective capacity to inhibit MCF7 growth when compared to the DMSO control cells. A growth inhibition benchmark of 88% set by the CAPE lead structure was also attained by esters **4**, **7**, **9**, **10**, **12**, **13** and **17**.

Given that CAPE is well-documented as an efficient inducer of apoptosis, we explored whether the observed growth suppression mediated by our CAPE derivatives was a result of caspase-dependent apoptosis. We thus treated the MCF7 breast cancer cell line with 10 µM of our CAPE derivatives and monitored caspase 3/7 activity at 1, 3 and 5 days post-treatment (Figure 2B). As expected, CAPE revealed a good capacity to induce apoptosis through a progressive induction of caspase 3/7 activity over the period of 5 days of treatment. Interestingly, we observed that many CAPE derivatives also induced strong apoptotic events when compared to vehicle solvent-treated control cells. In the interest of characterizing new, effective CAPE-derived compounds as anticancer agents, we selected the top five compounds capable of inducing breast cancer apoptosis (*i.e.*, **4**, **10**, **12**, **13**, and **17**) for further biological elucidation. Accordingly, most of the latter compounds demonstrate greater apoptosis-inducing ability in comparison to CAPE, especially at 48 h post-treatment.

### 2.2. Modulation of Cell Fate Signaling by Selected CAPE Analogs in Breast Cancer Cells

**Figure 3:**
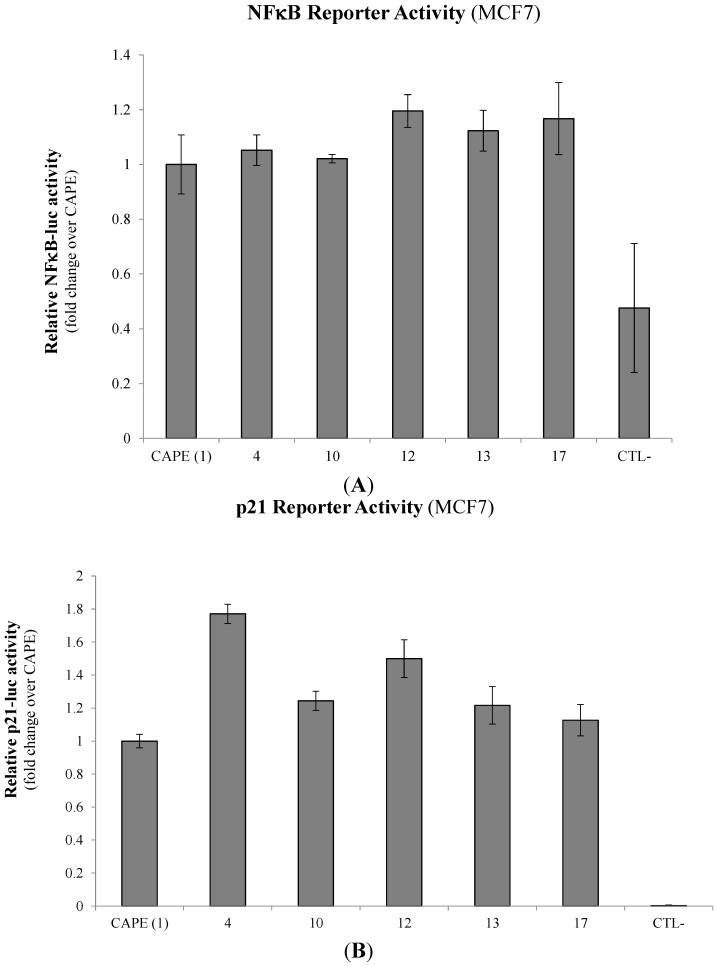
Modulation of cell fate regulators by selected CAPE analogs in breast cancer cells. MCF7 breast cancer cells were treated with 10 µM of selected compounds and evaluated for (**A**) NFκB and (**B**) p53 transactivation potential using NFκB-luciferase and p53-responsive p21-luciferase reporter gene constructs respectively. Relative light units (RLU) from normalized luciferase activity were then plotted in fold-change activity over the parent core CAPE compound. Control samples include the treatment with an IKK inhibitor (IKK-2 Inhibitor IV/10 µM) used as a positive control. Data is represented as means ± SEM of three experimental replicates.

It has been well-established that CAPE is a potent inhibitor of NFκB, a pivotal regulator of cancer cell growth and survival [18,19,23,24]. We thus set out to evaluate the capacity of selected CAPE derivatives to suppress NFκB transactivation using an NFκB-responsive luciferase-based reporter gene transfected into the MCF7 breast cancer cell line. Interestingly, none of the CAPE analogs tested (**4**, **10**, **12**, **13** and **17**) demonstrated any significant differences in their respective capacity to modulate NFκB-luciferase activity when compared to the CAPE lead compound (Figure 3A). The use of an IKK inhibitor (IKK-2 Inhibitor IV/10 µM) as a positive control however, inhibited NFκB-luciferase activity up to 53%.

We next wanted to explore the compounds’ ability to modulate other cancer cell pathways that could potentially explain their capacity to modulated breast cancer cell viability and apoptosis. Given that CAPE has previously been shown to activate the p53 pathway [18,22], we evaluated whether selected CAPE derivatives could induce p53 transactivation using a p53-responsive p21-luciferase reporter construct transfected into MCF7 cells. Interestingly, all selected CAPE analogs (**4**, **10**, **12**, **13** and **17**) induced greater p53 transactivation in MCF7 cells when compared to CAPE treatment alone (Figure 3B). More notably, esters **4** and **12**, with a propyl or phenylpropyl group, induced a ~1.75- and ~1.5-fold increase, respectively, over CAPE treatments.

### 2.3. Evaluation of p53-Mediated Apoptosis by CAPE Analogs in Breast Cancer Cells

**Figure 4:**
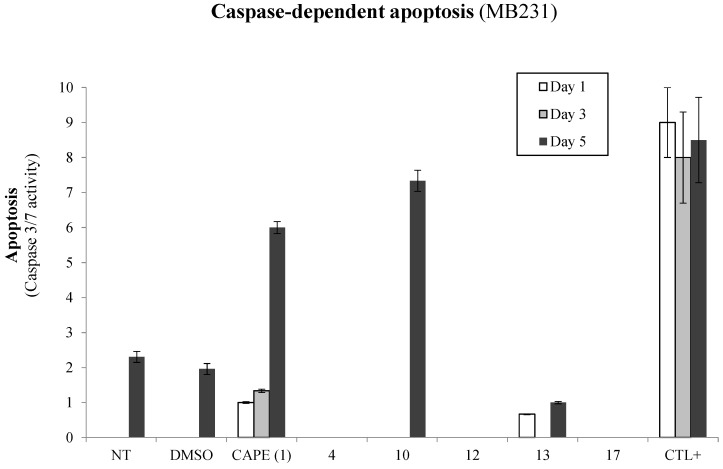
p53-Dependent apoptosis by CAPE derivatives in breast cancer cells. MB231 breast cancer cells were treated with 10 µM of selected compounds and incubated over time (24, 72 or 120 h) and evaluated for caspase-dependent apoptosis (Apo-ONE, Promega). Control samples include non-treated (NT) and solvent (DMSO) treated cells in addition to cells treated with apoptosis-inducing agent Melphalan (5 µM) as a positive control (CTL+). Results and standard deviations are representative of biological and experimental triplicates where the data is presented as the means ± SEM.

Given that the selected CAPE analogs (**4**, **10**, **12**, **13** and **17**) elicit greater p53 transactivation than the CAPE parental compound, we set out to determine whether the latter gain of p53 activity accounts for greater apoptosis potential in breast cancer cells. We thus made use of the MB231 aggressive breast cancer cell line, which lacks a functional p53 due to mutation. MB231 cells were treated with 10 µM of selected CAPE analogs and monitored over time (days 1, 3 and 5) for caspase-dependent apoptosis (Figure 4). Interestingly, all CAPE analogs, with the exception of CAPE itself and **10**, lost their ability to induce apoptosis in MB231 cells. These results suggest that CAPE and **10** compounds may mediate apoptosis predominantly through a p53-independent pathway.

### 2.4. Antioxidative Potential of CAPE Analogs in Breast Cancer Cells

It has been shown that apoptosis can also be induced by the alteration of the cell’s redox potential [25,26]. We therefore explored the capacity of the selected CAPE derivatives to elicit antioxidant activity as free radical scavengers. As depicted in Table 1, we observed that compounds **17** and CAPE displayed the highest antioxidant activity with IC_50_ values of 18.5 and 16.5 µM, respectively. These observations suggest that the redox modulation of CAPE, and more specifically **10**, may account for their potency to induce apoptosis through an extrinsic pathway.

**Table 1:** Antioxidant activity of selected CAPE derivatives.

Compounds	IC_50_ ± SEM (µM)
CAPE ^1^	16.5 ± 4.0
4	14.2 ± 1.7
10	12.5 ± 2.6
12	11.9 ± 0.6
13	12.8 ± 1.1
17	18.5 ± 2.1

^1^ Antioxidant activity as free radical scavengers of CAPE and its derivatives expressed as IC_50_. Values are means of 2 independent experiments, each performed in triplicate.

### 2.5. Discussion

As with many naturally derived compounds, CAPE has been extensively studied to elucidate its health benefits. Recently, this compound has seen a surge in interest as a potent anticancer drug [17,18,27]. In fact, CAPE has already been shown to inhibit breast cancer growth, while preserving the integrity of non-tumorigenic mammary epithelia cells [17]. However, in the interest of developing more efficient and targeted cancer treatments, it is crucial to explore new chemically derived compounds which could ultimately produce analogs with higher anti-cancer activity. More specifically, pharmacological properties with apoptosis-inducing capabilities are highly sought after, as they provide an effective, non-inflammatory approach to eliminating cancer cells to regain tissue homeostasis [28].

In this study, we explore the anticancer properties of 19 compounds, including the naturally derived CAPE as well as 18 novel synthetic analogs—17 esters (**2**–**18**) and one amide (**19**). We observe that CAPE derivatives effectively suppress breast cancer cell viability, with some of the esters showing greater inhibition of MCF7 breast cancer cell growth than CAPE itself and its amide analog (**19**). Given that apoptosis malfunction is a key hallmark of cancer development and tumor-cell survival, we evaluated the capacity of our panel of CAPE analogs for caspase 3/7 apoptosis activity in MCF-7 cells and observed that many derivatives (*i.e.*, **1**, **4**, **10**, **12**, **13** and **17**) induce strong caspase-dependent apoptosis events either equal to, or greater than, the lead reference compound. These findings are in agreement with previous reports demonstrating the potency of CAPE as an inducer of apoptosis [18,29,30,31,32]. Although we do not have a general trend, the replacement of the phenethyl moiety of CAPE with a propyl (**4**) or a phenyl (**10**) seems to be favorable for triggering apoptosis in MCF7 cells compared to CAPE lead structure. Moreover, the presence of a fluorine (**13**) or the replacement of the phenyl of CAPE by a biphenyl (**17**) or by a benzyl (**12**) appear to be favorable for apoptosis.

In our attempt to elucidate the mechanisms of action for CAPE analog-mediated apoptosis in breast cancer cells, we selected the top five apoptosis-inducing compounds for further biological characterisation. First, we wanted to assess the capacity of novel CAPE analogs to modulate cell survival pathways mediated by NFκB which could counterbalance the outcome of breast cancer cell fate. NFκB not only promotes neoplastic transformation but also activates anti-apoptotic proteins and signaling (reviewed in [2,5]). However, we did not see any significant changes in their respective capacity to modulate NFκB activation when compared to CAPE alone. These observations strongly suggest that: (1) the core structure of the lead CAPE compound is likely responsible for CAPE’s reported ability to suppress NFκB activity [18,33]; and (2) structure modifications did not account for changes of the NFκB survival pathway.

We next explored the capacity of the compounds to activate the p53 pathway, which has been previously demonstrated by CAPE [18,34]. P53-mediated signaling commonly induces caspase-dependent apoptosis, proving to be a potent target during cancer-associated pathway deregulation [6]. Mechanistically, cancer cells profit from these molecular cascades to promote tumor growth and aggressivity. Interestingly, all selected CAPE analogs tested demonstrated greater potential to induce p53 transactivation when compared to CAPE. We observed that the replacement of CAPE's phenethyl moiety by either a propyl (**4**) or a phenyl (**10**) induces p53 tumor suppressor activity which would likely lead to cancer cell apoptosis sensitivity. In addition, the presence of a fluorine (**13**) or, the replacement of CAPE’s phenyl by either a biphenyl (**17**) or a benzyl (**12**) also results in p53 induction which would be favorable for triggering apoptosis. We next wanted to define whether the gain of p53 activity accounts for the compounds’ apoptotic inducing ability using the MB231 p53-mutated breast cancer cell line [35]. Surprisingly, all tested compounds lost their ability to induce apoptosis with the exception of CAPE and **10**, thus suggesting that **4**, **12**, **13** and **17** may induce apoptosis in a p53 dependent manner. On the other hand, CAPE and **10** may alternatively make use of the extrinsic apoptosis pathway. These observations correlate with studies conducted by Nomura *et al.* (2001) which demonstrate the ability of CAPE to induce carcinoma cell apoptosis in both a p53-dependent and independent manner [22]. This being said, the structural modifications or similarities between CAPE, **10** and the other derivatives may define apoptotic signaling through either a p53-dependent or independent cascade. Compared to CAPE, **4**, **10**, **13** and **17**, as a phenyl ester, **10** is the least flexible of the series. These findings are of particular relevance when considering that inactivation or mutation of the p53 tumor suppressor gene is the most common alteration found in human cancers (up to 64%) (reviewed in [6]). Lead compound modifications such as **10** in this case could represent an effective anti-tumor strategy in cancers bearing p53 mutations.

Recently, research in p53-independent apoptosis has garnered interest to ultimately provide new therapeutic opportunities for many cancers [36]. p53-independent apoptosis, or the extrinsic apoptotic pathway, is usually induced by the binding of ligands to their cognate death receptors from the tumor necrosis factor receptor (TNFR) gene superfamily such as Fas/Fas ligand, TNF/TNF receptor [37,38,39], or less traditional death ligand/receptor interactions such as Apo2L/DR4, Apo2L/DR5 or Apo3L/DR3 [40,41,42,43]. These interactions eventually lead to the activation of effector caspases (caspases-3, -6 and -7) resulting in DNA fragmentation. NFκB activation also regulates cell death via the regulation of TNFR extrinsic factors which result in p53-independent cell apoptosis [44,45]. Accordingly, CAPE has previously been shown to induce extrinsic apoptosis through Apo2L/DR4 and DR5 receptors as well as Fas/Fas ligand interactions [18,46,47].

Another possibility is that **10** could mediate apoptosis through oxidative stress and the production of reactive oxygen species (ROS). ROS is a collective term that broadly describes O_2_-derived free radicals that can induce extrinsic apoptosis through the activation of JNK or attack DNA directly (reviewed in [25,26]). We and others have demonstrated the potential of CAPE structures to modulate the cell’s redox state [48,49,50]. We also observe that **10** is among the weakest antioxidants in our panel of analogs, suggesting a pro-oxidative potential that sensitizes cancer cells to extrinsic apoptotic events. A study by Choi and colleagues (2007) brings support to this latter hypothesis by demonstrating that CAPE sensitizes astrocytoma cells to Fas-induced apoptosis in a redox-dependent manner [51]. Alternatively, extrinsic apoptotic mechanisms could be at play to elucidate the potency of CAPE and **10** treatments in the induction of breast cancer cell apoptosis. The paucity of information in regards to the specific mechanism of action associated to CAPE analogs such as **10** in p53-independent apoptosis warrants further investigation as a potential anticancer drug.

## 3. Experimental Section

### 3.1. CAPE Analog Synthesis

As recently reported [52], alkyl **2**–**9** and aryl esters **10**–**18** were synthesized by esterification with selected alcohol and caffeic acid or acetylated caffeic acid. Amide **19** was synthesized from 2-phenylethanamine and acetylated caffeic acid. De-*O*-acetylation of the precursors of **10**–**18** and **19** resulted in the desired caffeic acid derivatives (Figure 1). The substituents were selected for their electronic and steric properties. To explore the effects of flexibility, addition of an insaturation (**8**) as well as the modification of the alkyl linker length (**10**, **11**, **12**) were investigated. The effects of additional phenyl moiety (**17**, **18**) as well as electron withdrawing and donating *p*-substituents (**16**, **17**) were investigated.

### 3.2. Cell Culture and Treatments

MCF7 and MDA-MB-231 (MB231) breast cancer cell lines were obtained from American Type Culture Collection (ATCC, Manassas, VA, USA). The cells were cultured in Dulbecco’s Modified Eagle’s Medium (DMEM) supplemented with 10% fetal bovine serum (FBS), 2 mM l-glutamine and 0.01 mg/mL of recombinant human insulin (MCF7). Cells were maintained in exponential growth and at 37 °C with 5% CO_2_. DMEM was obtained from HyClone (Thermo Scientific, Rockford, IL, USA), FBS from PAA Laboratories (Etobicoke, ON, Canada) and other reagents from Sigma-Aldrich (St. Louis, MO, USA).

Treatments consisted of CAPE derivatives reconstituted in DMSO at the indicated concentrations and incubated for the specified time points with cells seeded in 96-well microplates. In the specified experiments, cells were submitted to chemical inducers of caspase-dependent apoptosis (Melphalan/5 µM) or inhibitors of NFκB (IKK-2 Inhibitor IV/10 κM) used as controls.

### 3.3. Cell Viability and Apoptosis Assays

5 × 10^3^ cells were seeded in 96-well plates and analyzed at the indicated time points for cellular viability and apoptosis using multiplex assays CellTiter Blue^®^ and Apo-ONE^®^ kits respectively (Promega, Madison, WI, USA) according to the manufacturer’s instructions. In brief, 20 µL of CellTiter Blue^®^ substrate was added to 100 µL of media containing the cells and incubated at 37 °C for 1 h. Then, the microplates were subjected to analysis on a fluorescence microplate reader (FLUOstar Optima, BMG Lab technologies, 544_Ex_/590_Em_). Apoptosis was then measured on the same microplate by removing 80 µL of the total media and adding 40 µL of the Apo-ONE^®^ substrate. Next, the microplate was incubated at room temperature for 1h on a plate shaker and analyzed by fluorescence reading (485_Ex_/520_Em_).

### 3.4. Cell Transfections and Luciferase-Based Reporter Assay

Transfections were carried out using the XtremGENE reagent (Roche, Branford, CT, USA) according to the manufacturer’s guidelines. Briefly, cells were seeded in six-well plates 24 h pre-transfection at a density of 3 × 10^5^ cells/well. Cells were then incubated with a DNA-reagent complex (ratio of 2 μg of DNA/5 µL reagent) for 24 h in OPTI-MEM in reduced serum without antibiotics. Luciferase-based reporter gene assays were conducted using the Dual-Glo luciferase system (Promega) as described previously [53]. Briefly, cells were transfected with 2 µg of either a NFκB-luciferase construct [54]; or, a p53-responsive p21 promoter luciferase gene construct [55], lysed and analyzed for luciferase activity using a luminometer (BMG Fluostar, Fisher Scientific, Ottawa, ON, Canada). Relative reporter activity was calculated using experimental triplicates.

### 3.5. Radical Scavenging Activity

To determine the antioxidant activity of CAPE analogs, we measured their ability to reduce free radicals. The radical scavenging activity of CAPE derivatives was measured using a 2,2-diphenyl-1-picrylhydrazyl-(DPPH)-based radical generating system as previously described [56]. This assay measures the capacity of compounds to reduce free radicals through an electron transfer (ET) mechanism [57]. Controls within an optical density range of 0.350–0.360 at 520 nm were considered acceptable to avoid variations in the calculation of IC_50_ values. A solution of 60 mM DPPH (1 mL in ethanol) was mixed with 1 mL (in ethanol) of increasing concentrations of each CAPE derivative or with ethanol alone. Each mixture was then shaken vigorously and kept in the dark for 30 min at room temperature, after which the absorbance of DPPH at 520 nm was measured.

## 4. Conclusions

Altogether, our findings further validate the efficacy of CAPE as a potent anticancer agent as well as putting forth new CAPE-derivative compounds that show promising anticancer activity. Amongst these esters, we present their respective apoptotic inducing activities in both p53 competent and p53 mutant breast cancer cell lines. We strongly believe that CAPE, as well as possible derivatives including those shown here, warrant further investigation to create the possibility of adapting such analogs into promising therapeutic anticancer regimes.

## Abbreviations

**CAPE**:	Caffeic Acid Phenylethyl Ester
**NFκB**:	Nuclear Factor of kappa B Cells
**p53**:	tumor suppressor gene p53
**IκB**:	NFκB inhibitor
**IKK**:	IκB kinases
**DMSO**:	Dimethylsulfoxide
**µM**:	micromolar
**IC_50_**:	Half maximal inhibitory concentration
**TNF**:	tumor necrosis factor
**TNFR**:	tumor necrosis factor receptor
**Apo/DR**:	Trail receptor or Death receptor
**ROS**:	reactive oxygen species
